# Effects of phylogenetic distance, niche overlap and habitat alteration on spatial co-occurrence patterns in Neotropical bats and birds

**DOI:** 10.1098/rspb.2024.1679

**Published:** 2025-07-30

**Authors:** Anikó B. Tóth, John Alroy, S. Kathleen Lyons, Andrew Paul Allen

**Affiliations:** ^1^ Centre for Ecosystem Science, School of Biological Earth and Environmental Sciences, UNSW, Sydney, New South Wales 2052, Australia; ^2^ UNSW Data Science Hub, UNSW, Sydney, New South Wales 2052, Australia; ^3^ School of Natural Sciences, Macquarie University, Sydney, New South Wales 2109, Australia; ^4^ Department of Biological Sciences, University of Nebraska-Lincoln, Lincoln, NE 68588-0118, USA

**Keywords:** competitive exclusion, habitat alteration, interspecific spatial associations, phylogenetic niche conservatism, environmental filtering, community assembly, co-occurrence

## Abstract

Ecological interactions influence which species can coexist locally, but assessing the effects of interactions on species distributions at landscape to regional scales has proven challenging. Here, we present a new statistical method to address this question at the assemblage level. Our method, which we call mixed-effects co-occurrence modelling, entails fitting pairwise species co-occurrence data to generalized linear mixed models using Fisher’s non-central hypergeometric distribution. We use this method to examine the effects of phylogenetic relatedness, dietary-niche overlap (a proxy for potential food competition) and human-induced habitat alteration on pairwise co-occurrence patterns for bat and bird assemblages that span most of the Neotropics. For both assemblages, average pairwise co-occurrence increased with phylogenetic relatedness, indicating that phylogenetic niche conservatism contributes to environmental filtering at broad spatial scales. After controlling for phylogeny, variance in co-occurrence tended to be highest for species pairs in the same dietary guilds, suggesting varied responses to food competition. Effects of habitat alteration were relatively weak and inconsistent, though our analysis precluded identifying effects that were phylogenetically structured. Overall, our findings indicate that phylogenetic relationships among species pairs are instrumental in determining patterns of species co-occurrence and thereby influence how biological interactions play out at broad spatial scales.

## 1. Introduction

Biological interactions are essential for maintaining biodiversity [1,2] and ecosystem functioning [3] because they directly affect which species can coexist locally [4]. At evolutionary time scales, there is growing evidence that local biotic interactions, such as competition and facilitation, influence community assembly [5–7] at regional to continental scales [8], and rates of diversification and extinction for entire clades [7,9,10]. Still, at ecological time scales, there is only limited empirical evidence that species interactions consistently influence landscape- and regional-scale distributions of species [11–13].

Theory predicts that the degree of phenotypic similarity among species is central to community assembly [4]. If two species share ecologically significant niche traits, such as diet, they are more likely to find the same habitat suitable for occupancy. At ecological time scales, local habitats will, therefore, tend to accumulate species with similar niches, an effect known as environmental filtering [14], unless one species is capable of competitively excluding others [15]. At evolutionary time scales, natural selection may facilitate co-occurrence through speciation [16] and by limiting the phenotypic similarity of species that would otherwise compete, resulting in resource partitioning [17]. Phylogenetic niche conservatism likely plays a primary role in mediating niche-based mechanisms because, holding other factors constant, species that are more closely related phylogenetically will also tend to be more similar ecologically [18] and to be in closer proximity geographically [19–21]. Consequently, there is growing recognition of the need to incorporate phylogenetic information when analysing species co-occurrence data [19,22].

Efforts to infer interaction mechanisms from spatial patterns of species co-occurrence at broad spatial scales have proven contentious [13,23–29] owing largely to challenges in defining null expectations for species distributions in the absence of biotic interactions [14]. Defining null expectations is difficult because species distributions are influenced by other deterministic processes besides species interactions, including biogeographical effects, phylogenetic constraints and environmental filtering [13], and by stochastic processes such as dispersal and environmental stochasticity [30]. Furthermore, statistical power is often deemed insufficient to infer interspecific interactions from patterns of co-occurrence for the vast majority of species pairs owing to low occupancies for most species [31].

The above-mentioned challenges have complicated the task of assessing the effects of human land use on species interactions. Nevertheless, we expect these effects to be important because humans have been implicated in homogenizing communities [32], even when species richness is unchanged [33,34], and in reducing species co-occurrences [35,36]. Owing to logistics, evidence that human activities alter species interactions has been gathered mainly at local scales on systems with only a few species (e.g. [36–40]). Studies documenting changing interactions for entire assemblages (e.g. plant–pollinator networks) are rare and difficult to generalize owing to inconsistencies in data collection methods and sampling effort [41]. Complicating matters, rapid environmental change can rewire biological interactions [42] without necessarily adding or removing species [43]. Thus, there is an urgent need to fill this gap in our understanding of how local interactions scale up to influence spatial distributions of species at broad spatial scales, and how human activities might alter community assembly processes.

Here, we present a new statistical method for analysing spatial distributions of species at the assemblage level. Our method, which we call mixed-effects co-occurrence modelling, entails fitting species co-occurrence data to Bayesian generalized linear mixed models using Fisher’s non-central hypergeometric distribution [44,45]. This distribution has one fitted parameter that quantifies the strength and direction of the interspecific spatial association for a species pair based on how the odds that one species will occur at a given site are related to the presence or absence of another species [46]. A negative association, or segregation, implies that the two species composing the pair co-occur less often than expected by chance, as would happen if the two species have non-overlapping geographical ranges or occupy distinct habitats, or if one species competitively excludes the other. On the other hand, a positive association, or aggregation, implies that the two species co-occur more often than expected by chance, as would happen through environmental filtering if the two species are capable of co-existence and have overlapping species ranges and similar niches, perhaps in part owing to shared evolutionary history. We use this modelling framework to examine the independent and interactive effects of phylogenetic relatedness, dietary-guild overlap (a proxy for potential food competition) and habitat alteration on pairwise co-occurrence patterns for Neotropical bats and birds.

## 2. Material and methods

### (a) Data

Site-level metadata (including habitat alteration status), species abundance data and species dietary-guild data for Neotropical bats and birds were downloaded from the Ecological Register (http://ecoregister.org) [47]. The sites in the register are classified into nine disturbance categories (none, cropland, pasture, plantation, secondary forest, fragment, disturbed forest, inhabited area and combined), which we collapsed into two (altered and intact) based on whether any alteration was recorded (i.e. any category other than ‘none’). Sites were chosen, as described in the electronic supplementary material, to ensure that the altered and intact habitat types were similarly distributed over Central and South America. A total of 53 altered and intact sites were included in our analyses for bats, and totals of 42 altered and intact sites were included in our analyses for birds (electronic supplementary material, figure S1). The bat and bird species occupancy data were, respectively, sourced from 90 and 71 published field studies. Species were matched to the Open Tree of Life (OTL) [48] and dropped if they had incomplete dietary or phylogenetic information. A total of 190 bat species and 1197 bird species met our criteria for inclusion in the analysis (see electronic supplementary material for further details). A summary of standard community metrics for each assemblage can be found in tables S1-S2.

We analysed every pairwise combination of species for each of the two assemblages. The total numbers of pairwise species combinations analysed were higher for the intact habitats (respective totals of 14 365 and 452 676 for bats and birds) than for the altered habitats (respective totals of 9591 and 264 628 pairs) because more species were observed in the intact sites (170 bat species and 952 bird species) than in the altered sites (139 bat species and 728 bird species). The dietary-guild data were used to assign each species pair to one of the three levels for dietary-guild overlap (levels: low, medium and high). Dietary-guild overlap serves as a proxy for potential food competition because, while pairs are assigned to categories based on broad dietary-guild assignments (e.g. invertivores), two invertivorous bird species can only compete for food if they consume the same invertebrate species. High-overlap pairs are defined as those assigned to the same primary and secondary dietary guilds, but we allowed the order of these to be reversed (e.g. a frugivore–nectarivore and a nectarivore–frugivore would be equated). Medium-overlap pairs share some, but not all, dietary-guild assignments (e.g. a frugivore and an invertivore–frugivore) and are, therefore, expected to have a lower potential for interspecific food competition than high-overlap pairs. All pairs that included an omnivore were placed in the medium-overlap group. Low-overlap pairs share no dietary-guild assignments (e.g. a nectarivore and an invertivore). They have negligible potential for interspecific food competition and therefore serve as our control.

Phylogenetic distance for each species pair was calculated based on the number of branch nodes separating the pair [49] using phylogenetic trees obtained from the OTL [48] with the R package *rotl* [50]. Phylogenetic distance serves as a proxy for other variables, besides dietary-guild overlap, that may influence co-occurrence and are phylogenetically conserved. For example, species that are more closely related phylogenetically may be more likely to compete for the same roosting sites. Prior to analysis, phylogenetic distance data were logged, centred and scaled to have a mean of 0 and a variance of 1 and then binned into five equal-length intervals. All pairs within a given bin were then assigned the average phylogenetic distance for all pairs within the bin. We refer to this quantity as the standardized phylogenetic distance. Binning the phylogenetic distance data reduces the time and memory requirements for model fitting by an order of magnitude by facilitating the pooling of paired co-occurrence data for analysis, as described in section (d).

### (b) Study design

We adopt a comparative approach for analysing spatial co-occurrence patterns using an appropriate co-occurrence metric (described in section (c)) for all pairwise combinations of species. We evaluate the independent and interactive effects of three distinct predictors on pairwise co-occurrence patterns: standardized phylogenetic distance, dietary-guild overlap (levels: low, medium and high) and habitat type (levels: altered and intact).

**Figure 1.**
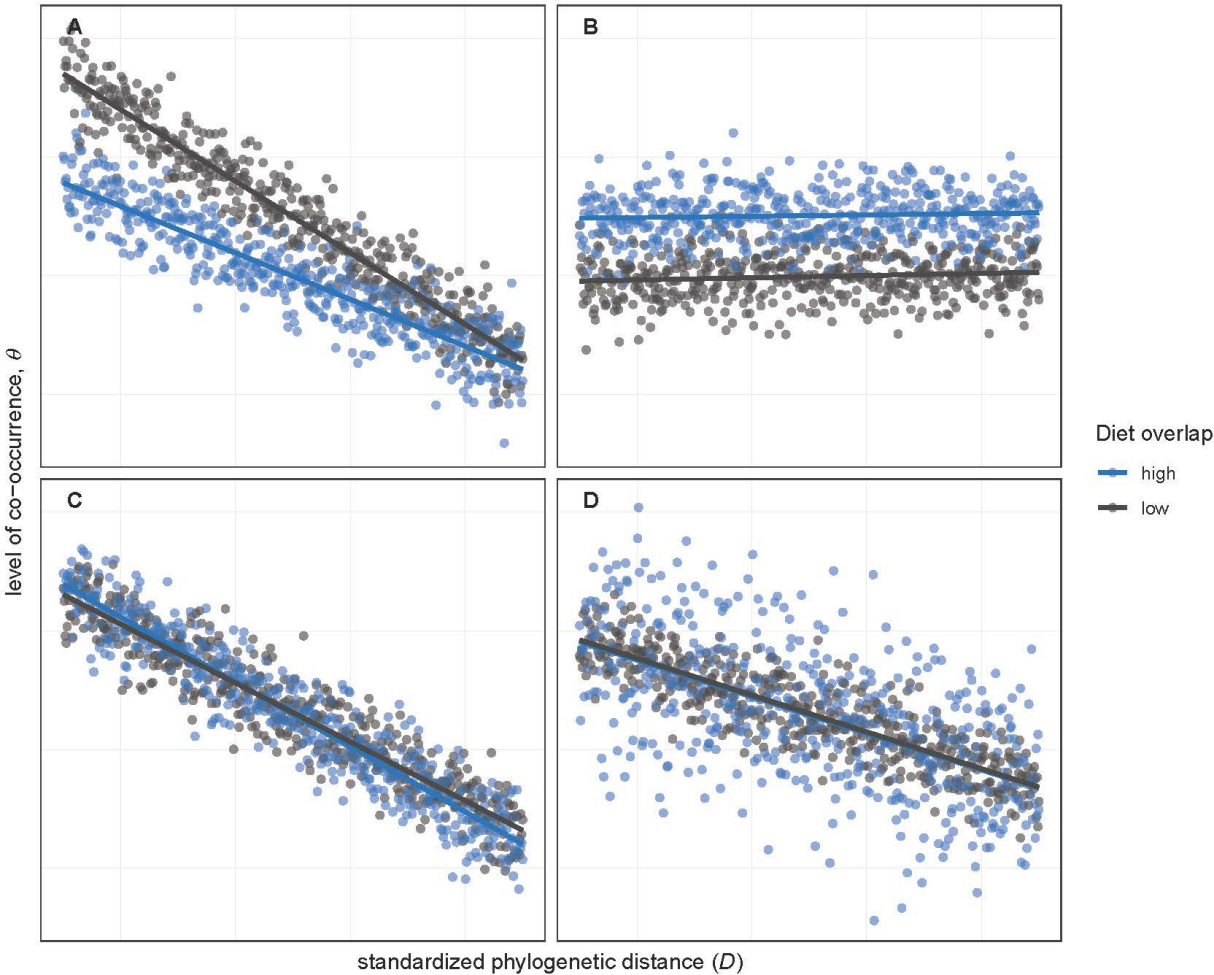
Conceptual figure demonstrating the independent and interactive effects of phylogenetic distance and dietary-guild overlap (high = blue versus low = grey) on spatial patterns of co-occurrence under four contrasting scenarios given our study design. First, a negative slope for phylogenetic distance (A,C,D) indicates that close relatives co-occur more frequently than distant relatives, suggesting that niche conservatism contributes to environmental filtering, while a slope near 0 (B) indicates a limited effect of niche conservatism. Second, slope differences among dietary-guild-overlap groups (A) indicate that the effects of niche-based mechanisms on co-occurrence vary depending on the degree of relatedness. Third, a depressed intercept for the high-overlap group as compared with the low-overlap group (A) suggests that competitive exclusion plays a primary role in determining co-occurrence patterns, whereas the opposite scenario (B) suggests that environmental filtering predominates. Finally, fourth, given parallel slopes (B–D), higher residual variance for the high-overlap group than the low-overlap group (D) suggests that diet similarity induces both resource partitioning and competitive exclusion in pairs with true diet overlap.

Under phylogenetic niche conservatism, species with closer phylogenetic relationships should also be more similar ecologically [18] and in closer spatial proximity. Consequently, a negative slope for the relationship of co-occurrence to phylogenetic distance would suggest that niche conservatism promotes environmental filtering (figure 1A,C,D), whereas a positive slope would indicate that it promotes competitive exclusion. A weak relationship would suggest that niche conservatism is of limited importance (figure 1B). Finally, an interaction between phylogenetic distance and dietary-guild overlap, which would manifest as slope differences among overlap groups, would indicate that niche-based processes are being moderated by phylogenetic relatedness (figure 1A).

Holding phylogenetic effects constant, we would expect average co-occurrence to be lowest for the high-overlap group if competitive exclusion plays a primary role in determining patterns of co-occurrence (figure 1A). On the other hand, if environmental filtering predominates, our expectation is that average co-occurrence will be highest for the high-overlap group owing to shared niches (figure 1B). If diet overlap has no effect, we expect the groups to show no differences (figure 1C). Finally, greater variance in co-occurrence for the high-overlap group as compared with the others would be expected if competitive exclusion and resource partitioning both occur in response to food competition but on a species-pair-specific basis (figure 1D). If habitat alteration shapes co-occurrence patterns through its effects on species interactions, we would expect there to be an interaction between the habitat variable and the dietary-guild-overlap variable.

### (c) Co-occurrence metric

Given that our study design entails a comparative approach, a metric is needed that quantifies the magnitudes and directions of spatial associations for species pairs. The fitted parameter θ of Fisher’s noncentral hypergeometric distribution (NHD) has these attributes [44]. We therefore assume that the number of sites of co-occurrence, $N_{\text{AB}}$, for a given pair of species, A and B, adheres to the NHD [45]


(2.1a) f(NAB|θ)=(NANAB)(N(A)NB−NAB)exp⁡(NABθ)P0(θ),

where $N_{A}$ and $N_{\left(A\right)}$ are the respective numbers of sites with species A present and absent and $N_{B}$ is the number of sites with species B present. The binomial coefficient nk=n!/k!/n-k! represents the number of ways to choose an unordered subset of *k* elements from a fixed set of *n* elements, so the product (NANAB)(N(A)NB−NAB) in the numerator of equation (2.1a) represents the number of ways that two species can achieve $N_{AB}$ co-occurrences given fixed values for the two species’ occupancies ($N_{A}$ and $N_{B}$) and for the total number of sites ($=N_{A}+N_{\left(A\right)}$). The denominator, $P_{0}\left(\theta \right)$, is calculated by summing the quantity calculated in the numerator over all possible $N_{AB}$ values [45], from $\max\left(0,N_{B}-N_{\left(A\right)}\right)$ to $\min\left(N_{A},N_{B}\right)$:


(2.1b) P0(θ)=∑n=max(0,NB−N(A))min(NA,NB)(NAn)(N(A)NB−n)exp⁡(nθ).

The NHD has one fitted parameter,


(2.1c) $$\theta =log\psi =log\left(\frac{\pi_{B|A}}{\pi_{B|\left(A\right)}}\right)=log\left(\frac{p_{B|A}\left(1-p_{B|\left(A\right)}\right)}{\left(1-p_{B|A}\right)p_{B|\left(A\right)}}\right),$$

which is the logarithm of an odds ratio $\psi =\pi_{B|A}/\pi_{B|\left(A\right)}$, where $\pi_{B|A}$ and $p_{B|A}$ are the respective odds and probability that species B occupies a site given that species A is present, and $\pi_{B|\left(A\right)}$ and $p_{B|\left(A\right)}$ are the respective odds and probability that species B occupies a site given that species A is absent. The value of θ and the probability of having $N_{AB}$ co-occurrences given θ, $f\left(N_{AB}|\theta \right)$ are both independent of which member of the species pair is arbitrarily chosen as species A. The NHD is typically parameterized using ψ rather than θ, but ψ has a lower bound of 0, making it less convenient for model fitting and biological interpretation.

The NHD parameter θ directly quantifies spatial patterns of co-occurrence for a species pair [44]. If θ<0, species B is less likely to occur at a site if species A is present. If θ>0, species B is more likely to occur at a site if species A is present. If θ=0, the two taxa are independently distributed. When θ=0, the NHD reduces to the standard (i.e. centred) hypergeometric distribution, which has been used as a null model to evaluate whether patterns of co-occurrence differ from random expectations [51].

We chose the NHD parameter θ as our co-occurrence metric over other options for several reasons. First, while θ is mathematically related to existing co-occurrence metrics (e.g. the mid-*P* variant of Fisher’s exact test [46]), it fundamentally differs in that it is not based on a null model. Instead, θ directly quantifies the magnitude of the association of one species’ presence on the odds of occurrence of another (analogous to the distinction between a correlation measure, *r*, and a *p*-value calculated under the null hypothesis that *r* = 0). Thus, θ is biologically interpretable (equation (2.1c)), and it is directly comparable between pairs. Finally, θ is expected to be largely insensitive to the number of sites in the study assemblage [44].

In the electronic supplementary material, we evaluate the ability of θ to quantify spatial associations between species. Our analysis highlights three general issues that arise when fitting the NHD to co-occurrence data:

**Figure 2.**
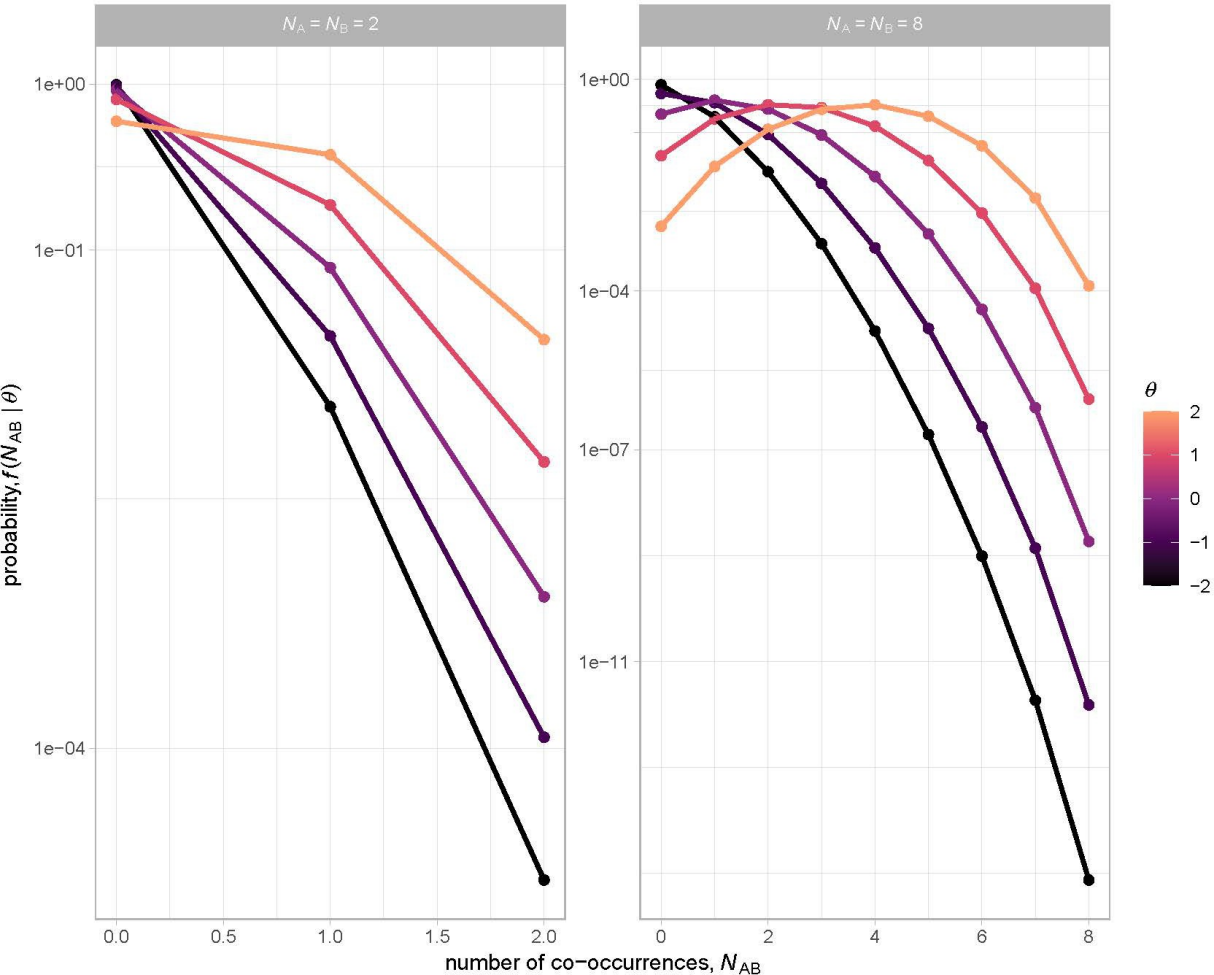
Effects of species occurrence (indexed by $N_{A}$ and $N_{B}$) and spatial aggregation (θ>0) and segregation (θ<0) on the probability distribution of co-occurrences demonstrate the performance of θ. Probabilities were calculated using equation (2.1) based on a total of $N_{A}+N_{\left(A\right)}=50$ sites.

(i) A non-overlapping pair (i.e. $N_{AB}=0$) will be observed with higher probability for a species pair with lower occurrence values ($N_{A}$ and $N_{B}$), irrespective of θ (figure 2).
(ii) An observation of a non-overlapping pair will not *by itself* be biologically informative about the value of θ because, in this case, the maximum likelihood estimate $\hat{\theta }=-$∞.
(iii) The amount of information about θ that can be inferred from a single observation is generally lower for a species pair with lower occurrence values (electronic supplementary material, figure S3).

To assess patterns of co-occurrence and their correlates at the assemblage level, we propose a new statistical method, mixed-effects co-occurrence modelling (MCM). Here we use MCM first to pool co-occurrence data for multiple species pairs into sets based on shared characteristics, and then to calculate θ estimates, and identify predictors of these estimates, for co-occurrence sets. It is through this process of pooling pairs into co-occurrence sets that issues (i)–(iii) are addressed.

### (d) Mixed-effects co-occurrence modelling

**Figure 3.**
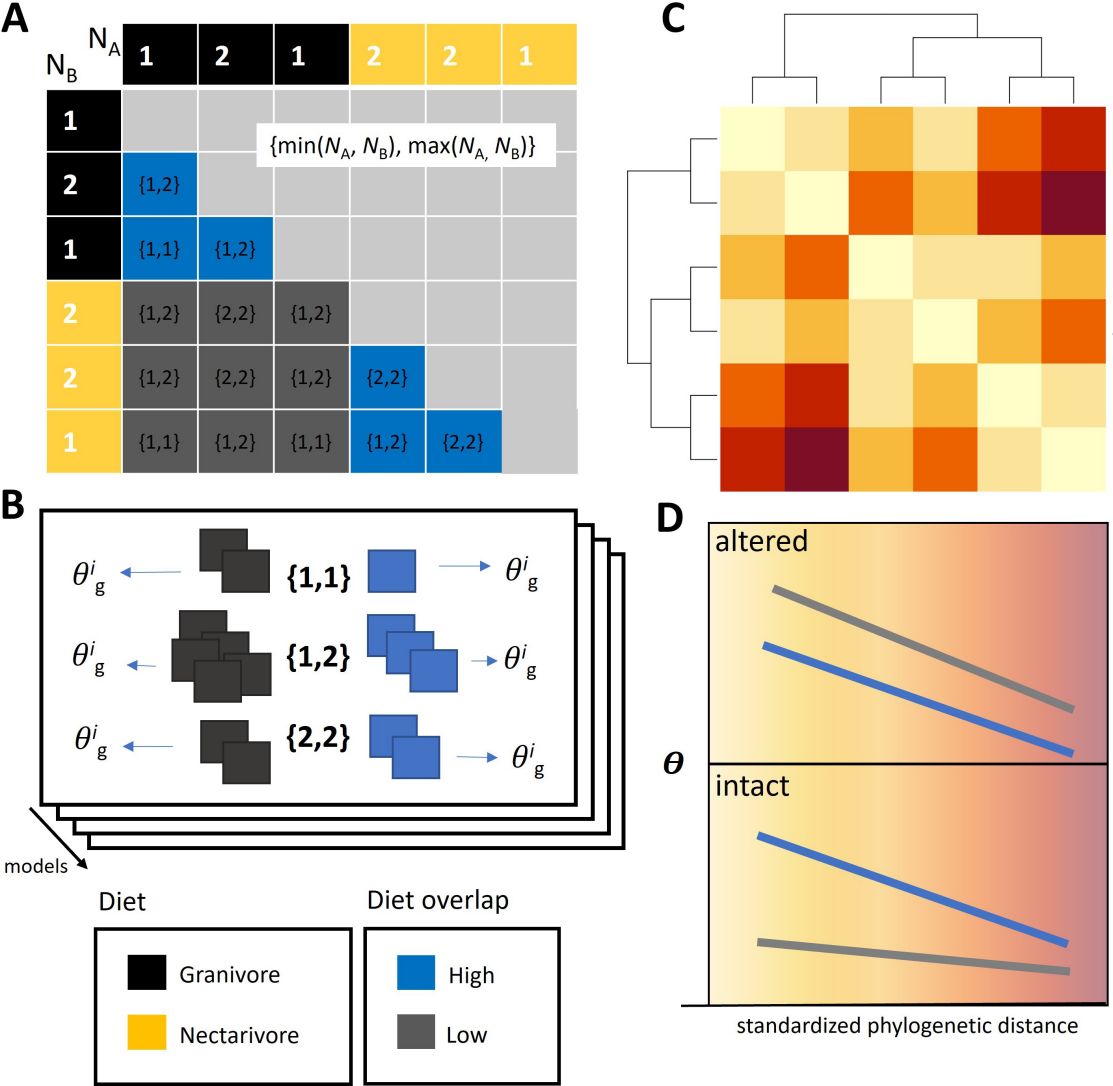
Conceptual figure demonstrating the experimental design. (A) Pairwise combinations of species (black and yellow cells corresponding to diet guilds) are depicted using an adjacency matrix. White numbers along the sides of the adjacency matrix are the occupancies $N_{A}$ and $N_{B}$ (i.e. numbers of occupied sites) for species A and B, respectively. Cells within the adjacency matrix (corresponding to one species pair each) are assigned to groups based on habitat (altered or intact) and/or guild overlap (low, medium, or high). The high- and low-overlap groups are, respectively, denoted by blue and grey cells in the panel. Species pairs within groups are then subdivided into co-occurrence sets, *i*, based on their paired occupancy values (black paired numbers, calculated as {$\min(N_{A},N_{B}),\max(N_{A},N_{B})$}) and other variables (e.g. diet-pairing granivore–granivore, nectarivore–nectarivore and granivore–nectarivore). (B) Estimates of spatial association are then calculated separately for the co-occurrence sets in each group, $\theta_{\text{g}}^{i}$, and averaged at the group level, $\overset{-}{\theta_{\text{g}}}$. (C) These θ estimates are refined by incorporating phylogenetic distances and trialling a series of models that use different rules for defining co-occurrence sets, represented by the stacked layers in (B). (D) Models are compared using the expected log predictive density, yielding a best-fit model with slopes, intercepts and θ variances that may differ among groups.

For the MCM analysis presented here, each species pair was assigned to a co-occurrence set based on a combination of levels for two or more of the following variables: standardized phylogenetic distance (five levels, corresponding to the five bins); dietary-guild overlap (levels: low, medium and high); diet pairing, which has levels defined based on all unique, unordered diet combinations (e.g. frugivore–frugivore, invertivore–invertivore and frugivore–nectarivore); habitat type (levels: altered and intact); occupancy pairing, which has levels defined based on all unique, unordered pair occupancies (i.e. {$min(N_{A},N_{B}),max(N_{A},N_{B})$}; see figure 3). Together, subsets of these variables define a new categorical variable, co-occurrence-set ID, which is used to assign each pair to a co-occurrence set.

For this analysis, occupancy pairing was always among the variables used to define co-occurrence-set ID because occupancy is known to be a primary determinant of the probability of co-occurrence [46]. For example, if each species occupies eight sites out of 50, a shift of θ from −2 (strong negative spatial association) to +2 (strong positive spatial association) will lead to a shift in the most likely co-occurrence value from 0 to 4 (figure 2). In contrast, if each species occupies two sites, this same shift in θ will leave the expected co-occurrence value at zero because species are too rare to be likely to co-occur. We pooled data into co-occurrence sets based on the number of occupancies (figure 3) rather than coarser occupancy bins (e.g. 1−5 sites, 6−10 sites, etc.). We did this because preliminary analyses indicated that, for our data, this resulted in the highest expected log pointwise (i.e. pair-level) predictive density, or expected log predictive density (ELPD), which is a measure of out-of-sample prediction accuracy that we estimated from the pointwise log likelihood using the R package *loo* [52].

To estimate θ for co-occurrence sets and identify predictors of variation in θ, we fit Bayesian mixed-effects models [53], treating co-occurrence-set ID as a random effect. It is by treating this variable as having a random effect on the intercept that pooling of pairs into co-occurrence sets occurs. More generally, in the parlance of mixed-effects modelling, we model the dependent variable, the number of co-occurrences, using an NHD distribution (equations (2.1a) and (2.1b)) with a log-link function for the NDH parameter ψ (equation (2.1c)), and we assume that θ ( = logψ) is influenced by both fixed and random factors. A random effect attributable to co-occurrence-set ID may arise through biological mechanisms, statistical considerations or some combination of the two. For example, a positive random effect for a co-occurrence set involving only the rarest taxa (occupancy pairing 1−1) may arise biologically if rare taxa are relatively poor competitors or may arise statistically if the θ estimate for the 1–1 occupancy pairing exhibits relatively little shrinkage towards the mean. We address this potential issue in the results by assessing whether the θ estimates vary systematically with the number of pairs in the co-occurrence set or with the occupancies of the rarer or more abundant species in the set.

Using the ELPD as our model selection criteria, fixed-effect predictors of θ were selected from among the following candidates: the quantitative variable standardized phylogenetic distance and the categorical variables dietary-guild overlap (levels: low, medium and high) and habitat type (levels: altered and intact). As is standard in mixed-effects models, random effects were assumed to be normally distributed with means of 0 (see electronic supplemetary material). As part of our analysis, we also considered diet pairing as a candidate random effect. This effect aims to capture residual differences in co-occurrence frequencies that remain after controlling for dietary-guild overlap. For example, if competition for food is more likely to result in competitive exclusion for frugivores than other high-overlap pairs, then we would expect the random effect for frugivore–frugivore to be negative after controlling for dietary-guild overlap.

Following the recommendation of Zuur *et al.* [54] for model selection, we first identified the optimal random-effects structure by fitting a series of models that included all possible fixed effects (i.e. main effects and interactions for standardized phylogenetic distance, dietary-guild overlap, and habitat), while allowing the random-effects structure to vary. We then identified the optimal fixed-effects structure by fitting a series of models that retained the optimal random-effects structure while varying the fixed effects. For both model-selection steps, we chose the structure yielding the highest ELPD [52]. The random-effects structure was varied by defining co-occurrence-set ID based on different subsets of variables from the list above, by assuming that the variance of this random effect was constant or varied with dietary-guild overlap or habitat and including or excluding diet pairing as an additional random effect (see electronic supplementary material, tables S4 and S6 for the full list of random-effects structures considered). The fixed-effects structure was varied by considering all possible subsets of the candidate predictor variables and their interactions (see electronic supplementary material, tables S5 and S7 for the full list of fixed-effects structures considered).

For each of the final models identified using the selection procedure described above, we performed two additional diagnostic analyses. First, to aid in assessing the relative importance of each fixed- and random-effect term in the final model, we compared the ELPD of the final model with that of each nested model with one fixed-effect term removed, and to that of a nested model with the standard deviation of the random effect on co-occurrence-set ID held constant rather than varying among groups. In the Results, we report these comparisons as *z*-scores, $z_{\Delta E}$=$\Delta E/s_{\Delta E}$, where ΔE is the ELPD of the full model minus the ELPD of the nested model and $s_{\Delta E}$ is the standard error of this difference. These model comparisons should not be viewed as formal statistical tests owing to non-independence issues that arise by including the occurrence data for each species in multiple pairwise comparisons.

Second, for each final model, we compared the posterior means of the parameter estimates with null distributions of the posterior means that were generated by refitting each final model to 100 randomized datasets. Randomized datasets were created by applying the curveball algorithm [55] to the binary site-by-species matrix. This algorithm uses sequential swaps to randomize the occurrences of species while preserving the observed species richness values for sites (i.e. the row totals in the matrix) and the observed numbers of occurrences for species (i.e. the column totals in the matrix). This procedure is undertaken without reference to the phylogenetic relationships or dietary-guild assignments of species, so disparity between the parameter estimates obtained from the actual and randomized data would indicate that biological mechanisms contribute to co-occurrence patterns. To ensure adequate randomization, each randomized matrix was produced using 10^8^ curveball swaps with the R package *vegan* [56].

We implemented our co-occurrence modelling framework by coding a custom distribution for the NHD using the R package *brms* [57], which provides an interface from R to the Stan probabilistic programming language [58]. To fit each model, we ran four Markov chain Monte Carlo (MCMC) chains of 3000 steps, including warm-up periods of 1000 steps and a thinning interval of 2, yielding posterior samples of size 4000. To ensure convergence, we inspected MCMC plots for the four chains and Gelman–Rubin statistics [53], which are calculated automatically by *brms*.

Changes in co-occurrence patterns between altered and intact habitats may arise owing to pairs changing their spatial relationships in response to modified interactions or through gains or losses of species. To investigate these possibilities, we fitted the models using data for all species (‘full’ models) and for the approximately 68% of bat species and approximately 42% of bird species that occur in both habitat types (‘restricted-pool’ models). Similar results for the full and restricted-pool models would suggest that differences in spatial associations between habitats are driven by changing spatial relationships rather than species pool differences.

## 3. Results

### (a) Bats

**Figure 4.**
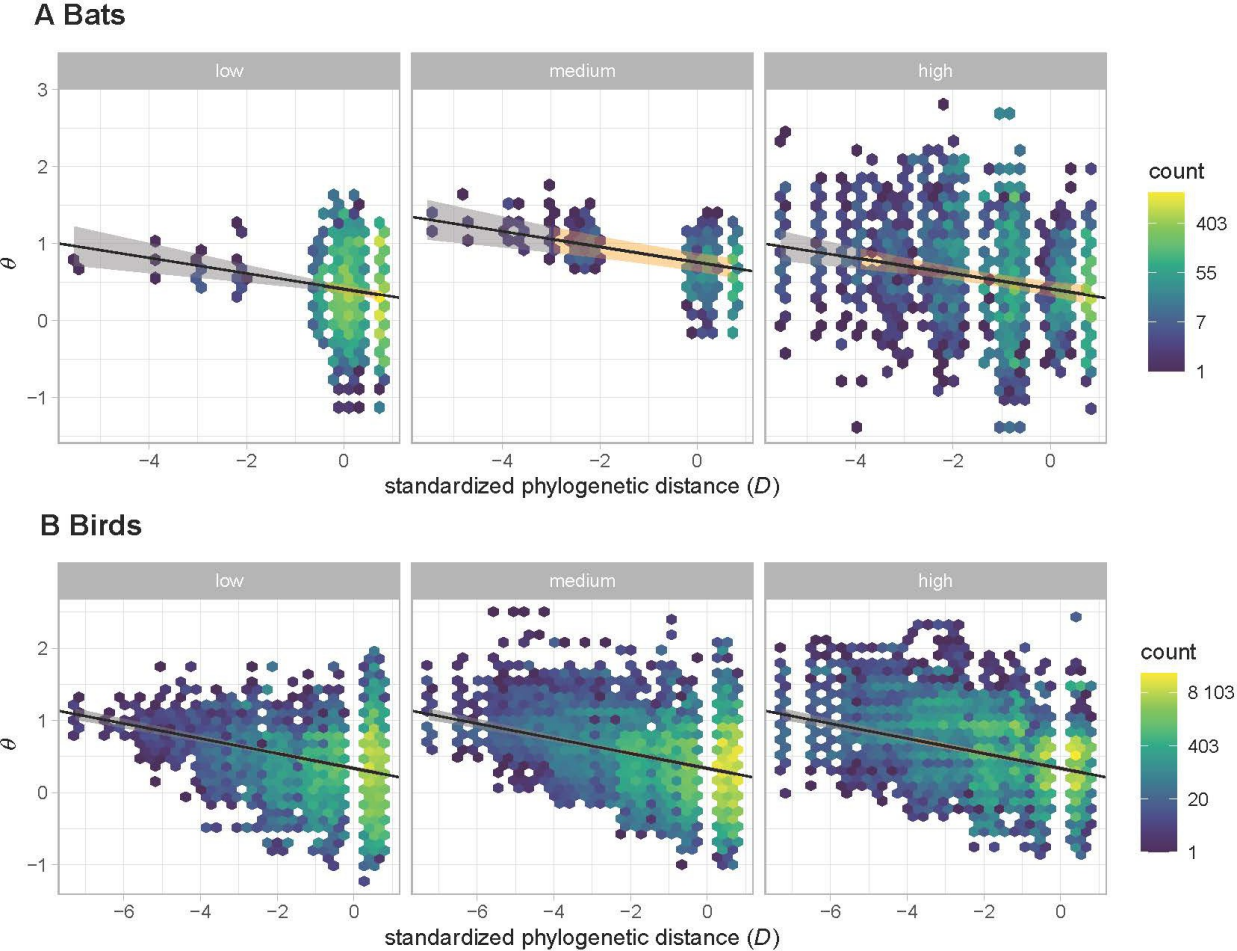
Relationship of the spatial association parameter *θ* to standardized phylogenetic distance for (A) bats and (B) birds in different diet-overlap groups. The *θ* estimates for co-occurrence sets are represented using a hexagonal grid, with colours to denote the numbers of pairs with overlapping estimates (note the logarithmic colour scale). The black lines represent the average posterior slope and intercept of each group, with shading used to represent 95% CIs for posterior predictions calculated based only on fixed effects. The colour of the CI envelope is orange inside and grey outside the 2.5 and 97.5% quantiles for standardized phylogenetic distances to emphasize the concentration of data at higher phylogenetic distances.

**Figure 5.**
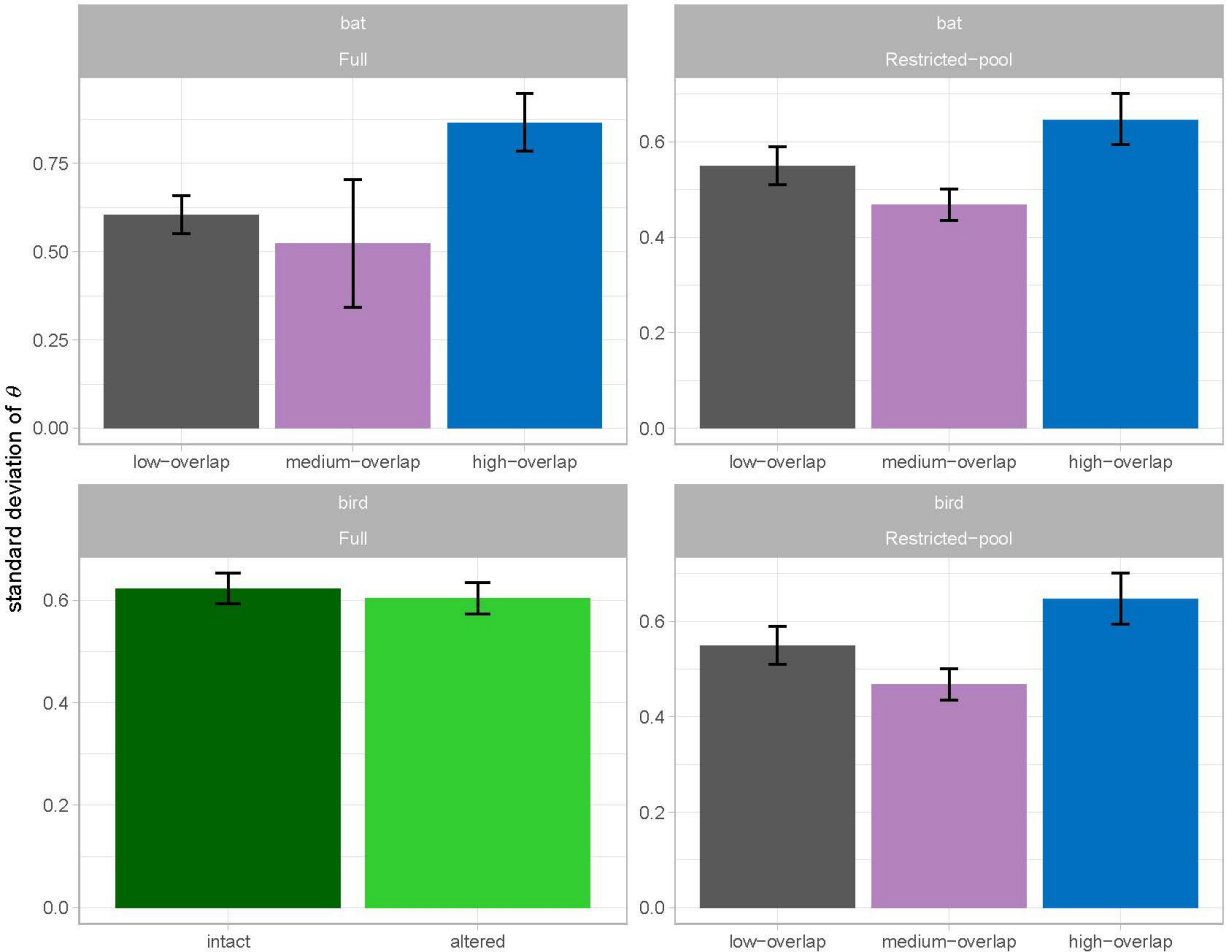
Standard deviations of θ for full (all species) and restricted-pool models (including only species found in both habitat types) for both taxa. The full bird model panel shows that standard deviations for the random effects vary by habitat, while the others vary by diet overlap, in accordance with our best-fit models. Error bars indicate 95% credible intervals.

For the full bat model (figure 4A), the optimal fixed-effects structure includes phylogenetic distance ($z_{\Delta E}$ = 4.00) and dietary-guild overlap ($z_{\Delta E}$ = 2.27) (electronic supplementary material, table S5). The slope for phylogenetic distance has a posterior mean of −0.10 (95% credible interval (CI): −0.14 to −0.06), implying that closely related bat species are more likely to co-occur than distantly related species (electronic supplementary material, table S3). At average phylogenetic distance, corresponding to a standardized distance of 0, the posterior mean for θ increases from 0.41 for the low-overlap (95% CI: 0.35–0.46) and high-overlap (95% CI: 0.36–0.52) groups to 0.75 for the medium-overlap group (95% CI: 0.61–0.88; electronic supplementary material, table S3). The optimal random effects structure includes one random effect on the intercept attributable to co-occurrence-set ID, which is defined based on unique combinations of levels for binned phylogenetic distance, diet pairing, habitat type and occupancy pairing (electronic supplementary material, table S4). After controlling for the fixed effects, the standard deviation of the θ estimates varies with dietary-guild overlap ($z_{\Delta E}$ = 2.99) and is higher for the high-overlap pairs (0.87; 95% CI = 0.79–0.95) than for the medium-overlap pairs (0.53; 95% CI = 0.35–0.71) and low-overlap pairs (0.60; 95% CI = 0.55–0.66) (figure 5).

The restricted-pool model for bats also includes phylogenetic distance ($z_{\Delta E}$ = 2.98) and dietary-guild overlap ($z_{\Delta E}$ = 2.31) as fixed effects (electronic supplementary material, table S9). The random-effects structure for this model is identical to that of the full bat model, including a standard deviation that varies with dietary-guild overlap ($z_{\Delta E}$ = 2.25) (electronic supplementary material, table S8) and is once again higher for the high-overlap pairs than for the medium- and low-overlap pairs (figure 5).

### (b) Birds

The full bird model (figure 4B) includes a single fixed effect attributable to phylogenetic distance ($z_{\Delta E}$ = 27.47; electronic supplementary material, table S7) with a slope of −0.10 (95% CI: −0.12 to −0.09; electronic supplementary material, table S3). So, just as in the bats, closely related bird species are more likely to co-occur than distantly related species. The model includes a single random effect on the intercept attributable to co-occurrence-set ID, which is defined based on the same subset of variables as in the two bat models (electronic supplementary material, table S6). Unlike in the bat models, during the first model-selection step, habitat was chosen over dietary-guild overlap as the optimal predictor of variation in the standard deviation for this random effect. However, after dropping all fixed-effect terms besides phylogenetic distance in the second model selection step (electronic supplementary material, table S7), an effect of habitat on the standard deviation of the random effect is no longer observed: the standard deviations of the θ estimates are essentially identical in intact habitats (0.62, 95% CI = 0.59 to 0.65) and altered habitats (0.60, 95% CI = 0.57 to 0.63) (figure 5), so the nested model with a constant standard deviation yields a better overall fit $(z_{\Delta E}$ = – 4.35).

The restricted-pool model for birds also includes phylogenetic distance as a fixed effect ($z_{\Delta E}$ = 3.89), along with habitat, which is of marginal importance ($z_{\Delta E}$ = 1.05) (electronic supplementary material, table S11). The model includes a single random effect on the intercept attributable to co-occurrence-set ID, which is defined based on the same subset of variables as in the three models above. However, unlike in the full bird model, in this model, dietary-guild overlap is selected as the optimal predictor of variation in the standard deviation ($z_{\Delta E}$ = 13.39), just as in the two bat models (electronic supplementary material, table S10). Moreover, after controlling for the fixed effects, the high-overlap pairs have a higher standard deviation (0.65, 95% CI = 0.59 to 0.70) than the medium-overlap pairs (0.47, 95% CI = 0.43 to 0.50) and low-overlap pairs (0.55, 95% CI = 0.51 to 0.59; figure 5), the same pattern we observed in both bat models (electronic supplementary material, table S3).

### (c) Model diagnostics

For all four of the final models described above, the residual variation in the θ estimates that remains after controlling for the fixed effects shows no systematic variation with the number of pairs in the set nor with the occupancies of the rarer or more abundant species in the sets (electronic supplementary material, figures S4–S6). These findings suggest that this variation is not a statistical artefact. The null-distribution analyses undertaken by fitting the final model structures described above to randomized data yield three noteworthy findings. First, the fitted intercepts obtained using the actual and randomized data are similar in magnitude (electronic supplementary material, table S3). Second, with the exception of fixed effects identified above as being marginal based on the ELPD differences (i.e. $z_{\Delta E}$< 2), the posterior means of the actual parameter estimates lie well outside the null distributions (electronic supplementary material, table S3). These findings suggest that the ELPD criterion was useful for model selection in this analysis despite the inherent non-independence of the co-occurrence data at the assemblage level. Third, the posterior means of the standard deviations for the random effects are consistently higher than those for the null distributions, suggesting that this variation is partly driven by biological mechanisms (electronic supplementary material, table S3).

## 4. Discussion

Our results for both the bat and bird assemblages indicate that closely related pairs co-occur more frequently than distantly related pairs (figure 4). These findings suggest that phylogenetic niche conservatism facilitates co-occurrence through its effect on environmental filtering [18]. Phyllostomid bat species exhibit varied morphologies [59] and share roosts [60], and closely related bat and bird species both display varied foraging strategies (e.g. [16,24,60–63]), which may help facilitate co-existence through micro-partitioning of food resources. Thus, despite well documented instances of interspecific aggression leading to competitive exclusion for some bird species (e.g. [24,64,65]), our findings suggest that coexistence among species that occupy similar niches is prevalent for both taxa. Importantly, however, fine, local-scale segregations may be lost owing to the spatial resolution of our samples [11].

The dominant role of phylogenetic relatedness as a driver of co-occurrence patterns likely reflects the large spatial extent of this study, which encompasses substantial turnover in species composition from Mexico to Argentina [66,67] (see electronic supplementary material, figure S2). A primary role for phylogeny as a driver of this turnover is supported by the observation that dispersal of nascent species is often limited in the Neotropics, resulting in spatial aggregations of closely related species [16]. Such aggregations may arise through allopatric or sympatric speciation mechanisms [20,21]. Here it is noteworthy that the intercepts of our fitted models, representing θ predictions at average levels of phylogenetic relatedness, were consistently >0 (indicating non-random patterns of co-occurrence), and of similar magnitude for both the actual and randomized co-occurrence data (approx. 0.3–0.4; electronic supplementary material, table S3). These findings could partly reflect the fact that the fixed-row–fixed-column approach we used to randomize the data is conservative [68] in that it retains some non-random biogeographical structures, particularly nestedness, by restricting allowable permutations of the binary site-by-species matrix [69]. Properties of the θ estimator may also contribute to the generally positive θ values we report. Specifically, given that the species occupancies in this study were consistently low relative to the total numbers of sites, and therefore that a co-occurrence value of zero is the most likely outcome over a broad range of θ values (figure 2), aggregated patterns of co-occurrence (corresponding to θ > 0) are more readily inferred statistically (see electronic supplementary material, figure S3). We therefore interpret the θ estimates comparatively by first partitioning variation in θ for co-occurrence sets among different effects, and then using randomizations to aid in establishing their importance, thereby using all available information [70]. Further work is needed to assess how spatial extent and resolution affect θ estimates through their influence on the fraction of non-zero entries in the binary site-by-species matrix (i.e. matrix fill).

Sharing of food sources appears to affect the spatial community structure of both taxa as well. Although we expected competition to exhibit variable patterns across various dietary pairs (e.g. if competition is stronger between nectarivorous than frugivorous pairs), this hypothesis was not supported because diet pairing was not incorporated as a random effect in any of our best-fit models for bats or birds. Nevertheless, in all except the full bird model, high-overlap pairs exhibit a greater variance of spatial associations than either low- or medium-overlap pairs (figure 5). This result suggests that competing pairs experience a range of species pair-specific drivers, from competitive exclusion to resource partitioning. Bats exhibit an additional diet-related effect. For both bat models, the medium-overlap pairs have higher average co-occurrence than the other two groups once phylogeny is accounted for (intercept of 0.75 versus 0.41—see Results). These unexpected findings are consistent with the hypothesis that the evolution of specialized feeding strategies in bats is more effective when there is a stabilizing niche difference that further reduces direct competition. Under this scenario, the advantage gained by resource partitioning is offset by the disadvantage incurred, on average, across pairs, when sharing space with species that have higher diet similarity.

Overall, our findings suggest that the effects of niche overlap can indeed be inferred from co-occurrence data if the effects of other mechanisms (e.g. phylogenetic niche conservatism) are accounted for and a control group (i.e. low-overlap pairs) is used as a benchmark for comparison. The use of a control group is a central tenet in experimental design, and yet it is seldom used in observational co-occurrence studies (but see e.g. [25]). Our finding that variance in co-occurrence tends to be higher for species with more similar diets is consistent with the argument that competitive exclusion is a common phenomenon, detectable from distribution patterns alone [23–25], among bird pairs that share dietary guilds [71] or are closely related [24]. While others have argued that bird pairs have statistically weak spatial associations that cannot be used to distinguish the effects of competition [26–28] from those of non-biological geographical mechanisms [23], these studies all used classical approaches to co-occurrence analysis that consider each species pair in isolation, reducing statistical power, particularly for rare taxa. Our proposed approach aims to help overcome this limitation by pooling pairs into co-occurrence sets for analysis. Addressing this issue is important given that rare taxa generally compose the vast majority of species, especially in tropical regions where competition is often presumed to be most intense [16].

While our results yield no compelling evidence that habitat alteration influences co-occurrence patterns, there are two issues worth bearing in mind. First, our results do not preclude the existence of habitat-alteration effects that are phylogenetically structured because controlling for phylogeny, as we do in our analysis, may obscure such effects. Second, our dataset includes multiple types of habitat alteration that were lumped into the single category ‘altered’ for this analysis. The majority of our altered sites (59%) are secondary or disturbed forests, which likely retain some of the complexity lost in more heavily disturbed environments like cropland or pasture. This may help to explain why habitat type was generally not identified as important in our analysis after controlling for phylogeny. Separately examining the effects of each type of habitat alteration using more extensive data might yield more nuanced results.

## 5. Conclusions

Our results demonstrate how basic functional and phylogenetic data, when applied to co-occurrence data using mixed-effects co-occurrence modelling, can be used to understand the interacting drivers of community assembly for speciose assemblages at broad spatial scales. We can partition the effects of dietary-guild overlap from those of phylogenetic relatedness and provide spatial association measures across all combinations of levels. Importantly, we do not simply note deviations from random expectations, but instead we quantify each effect, both singly and as interactions with other effects in our model set-up. We believe that our method has potential to improve understanding of community assembly, interactions and the maintenance of biodiversity across biological assemblages globally. Our results shed light on the relationship between ecological interactions, functional relationships and regional community assembly. In short, our approach helps pave the way for future research seeking to understand how community assembly emerges in response to various contexts and across spatial scales.

## Data Availability

All R workflows and data that support the findings of this study are freely available on GitHub at [72] and on Dryad at [73]. Supplementary material is available online [74].
